# Value of lymphadenectomy in patients with surgically resected pancreatic neuroendocrine tumors

**DOI:** 10.1186/s12893-022-01595-y

**Published:** 2022-05-10

**Authors:** Zheng Zhang, Fei Wang, Zheng Li, Zeng Ye, Qifeng Zhuo, Wenyan Xu, Wensheng Liu, Mengqi Liu, Guixiong Fan, Yi Qin, Yue Zhang, Xuemin Chen, Xianjun Yu, Xiaowu Xu, Shunrong Ji

**Affiliations:** 1 Department of Pancreatic Surgery, Fudan University Shanghai Cancer Center, 270 DongAn Road, Shanghai, 200032 People’s Republic of China; 2 Department of Oncology, Shanghai Medical College, Fudan University, Shanghai, People’s Republic of China; 3 Shanghai Pancreatic Cancer Institute, Shanghai, People’s Republic of China; 4 Pancreatic Cancer Institute, Fudan University, Shanghai, People’s Republic of China; 5 The First People’s Hospital of Changzhou, Changzhou City, Jiangsu Province People’s Republic of China

**Keywords:** Lymph node metastasis, Lymphadenectomy, Pancreatic neuroendocrine tumors

## Abstract

**Background:**

Although some factors that predict the prognosis in pancreatic neuroendocrine tumor (pNET) have been confirmed, the predictive value of lymph node metastasis (LNM) in the prognosis of pNETs remains conflicting and it is not clear whether regional lymphadenectomy should be performed in all grades of tumors.

**Methods:**

We included pNET patients undergoing surgery in Shanghai pancreatic cancer institute (SHPCI). The risk factors for survival were investigated by the Kaplan–Meier method and Cox regression model. We evaluated the predictors of LNM using Logistic regression.

**Results:**

For 206 patients in the SHPCI series, LNM was an independent prognostic factor for entire cohort suggested by multivariate Cox regression analysis. LNM (*P* = 0.002) predicted poorer overall survival (OS) in grade 2/3 cohort, but there is no significant association between LNM and OS in grade 1 cohort. Grade (*P* < 0.001) and size (*P* = 0.049) predicted LNM in entire cohort. Grade (*P* = 0.002) predicted LNM while regardless of size in grade 2/3 cohort.

**Conclusions:**

Based on our own retrospective data obtained from a single center series, LNM seems to be associated with poorer outcome for patients with grade 2/3 and/or grade 1 > 4 cm tumors. On the other way, LNM was seems to be not associated with prognosis in patients with grade 1 tumors less than 4 cm. Moreover, tumor grade and tumor size seem to act as independent predictors of LNM. Thus, regional lymphadenectomy should be performed in grade 2/3 patients but was not mandatory in grade 1 tumors < 4 cm. It is reasonable to perform functional sparing surgery for grade 1 patients or propose a clinical-radiological monitoring.

**Supplementary Information:**

The online version contains supplementary material available at 10.1186/s12893-022-01595-y.

## Background

Although pNETs are uncommon and their prognosis is better than that of pancreatic cancer, the incidence of pNETs is increasing [1, 2]. PNETs are heterogeneous neoplasms and can be divided into functional and non-functional according to hormone secretion [3]. Nonfunctional pNETs account for 80% of cases and radical surgery is the only way to cure resectable pNETs [3]. Unlike the obvious symptoms of functional pNETs, nonfunctional pNETs are either incidentally discovered by abdominal computed tomography or when symptoms associated with tumor compression or invasion become evident [4]. Although pNETs show indolent tumor biology, the 5-year survival rate of pNETs ranges from 15 to 100%. A subset of patients with aggressive tumors still has poor outcomes [5, 6]. Thus, identifying high risk factors for OS and making more appropriate management for these patients is urgent. Muscogiuri et al. aimed to exam how gender shapes risk factors with the hope of providing gender-tailored strategy [7]. Recently, family history of non-neuroendocrine gastroenteropancreatic (GEP) cancer, type 2 diabetes mellitus and obesity have been identified as independent risk factors for GEP-NENs by Feola et al. [8]. And in our study, we tried to explore the potential prognostic value in LNM.

At present, there are two international clinical staging systems for pNETs: the American Joint Committee on Cancer (AJCC) and European Neuroendocrine Tumor Society (ENETS) [9]. Although the two clinical staging systems are contradictory and inconsistent in data comparison, LNM is considered to be an important prognostic indicator by both of them and LN status includes N0 (no regional LNM) and N1 (regional LNM) [10]. However, recommendations for lymphadenectomy in pNETs are still inconsistent. The National Comprehensive Cancer Network (NCCN) guidelines recommend regional lymphadenectomy for tumors of 1–2 cm due to the risk of LNM while performing routine lymphadenectomy blindly is not advocated in all tumors of < 2 cm [11].

The literature addressing the significance of LNM in the management of nonfunctional pNETs remains conflicting. Several studies have demonstrated that LNM was not independently correlated with survival [12, 13] while others have suggested that LNM is associated with poorer overall survival (OS) or disease-free survival (DFS) [14, 15]. Therefore, a more accurate classification of these patients based on LNM is needed. The Union for International Cancer Control and AJCC tumor, node, metastasis (TNM) classifications divide LNM into N1 (1–3 positive LNs) and N2 (≥ 4 positive LNs) for high-grade pNET [16, 17]. Nevertheless, the distinction is not valid for well-differentiated pNETs. The accuracy in prognostication of a TNM staging system based on positive LNs for well and intermediately differentiated pNETs is unknown.

Given that prognosis of pNETs with LNM varies widely and the importance of differentiation for LNM in TNM classification, we evaluated the predictive value of LNM for prognosis based on tumor grade. Moreover, we studied the preoperative predictive factors for LNM to guide surgical procedures and avoid unnecessary lymphadenectomy.

## Methods

### Patients and data collection

Patients diagnosed with pNET pathologically (2012–2018) from SHPCI were enrolled in our study. They were classified based on the AJCC Staging Classification (8th edition) and divided into two groups (grade1 and grade 2–3). A monthly review of medical reports confirmed the follow-up data and we contacted the patients or their relatives to track disease progress, vital status, and date of death if applicable. The follow-up duration ranged from 3.63 to 128.67 months. The study was known and recognized by all the patients and passed the audit procedure of the Fudan University Shanghai Cancer Center Ethics Committee.

Inclusion criteria for this study included patients with a pathological diagnosis of pNETs and LN examination. Patients were included if they underwent pancreatic resection and had no distant metastasis. We included patients only if their tumor was > 1 cm and OS > 3 months to rule out randomness and perioperative mortality. We retrieved demographic details, including sex and age. Tumor variables included LNM, location, grade, functional status, and tumor size. Since the surgical method has not yet been determined in pNETs, we tended to discuss carefully the function preserving surgical method in Grade 1 tumors. Therefore, we divided the patients into grade 1 and grade 2/3 cohorts. To note, there were no neuroendocrine carcinoma (NEC) in grade 3 patients and all patients were with sporadic pNET [18, 19].

### Statistical analysis

All the data analyses were conducted by GraphPad Prism version 7.0 (GraphPad Software, San Diego, CA, USA) and SPSS version 25.0 (SPSS, Chicago, IL, USA). A two-sided *P* < 0.05 was considered statistically significant. Categorical variables were evaluated by χ^2^ or two-sided Fisher’s exact test. The preoperative variables predicting LNM were investigated using binary logistic regression. Cox proportional hazards regression analyses were performed to evaluate factors affecting OS by grade. The corresponding hazard ratios (HRs) and 95% confidence intervals (CIs) were calculated. The OS was calculated using Kaplan–Meier analysis and Log-rank test.

## Results

### Patient characteristics

The study included 206 patients with pathologically diagnosed pNET from the SHPCI, including 96 with grade 1 tumors and 110 patients with grade 2/3 tumors (Table 1). The number of harvested lymph node ranged from 1 to 26. The median follow-up for this cohort was 41.68 months. For patients with grade 1 and 2/3 tumors, the percentage of patients aged < 60 years was 76.0% (*P* = 0.871) and 63.6% (*P* = 0.838), respectively. There were 45 female and 51 male patients in grade 1, with no significant gender difference (*P* = 1.000). Also, no obvious difference between male (n = 69) and female (n = 41) was found in grade 2/3 cohorts (*P* = 0.932). Approximately 59.4% and 51.8% of patients had a tumor located at the body or tail of the pancreas in the grade 1 (*P* = 0.056) and 2/3 cohorts (*P* = 0.229). In grade 1 cohort, there were 72 tumors with smaller tumor size compared to 24 tumors that larger than 4 cm (*P* = 0.497). Whilst in grade 2/3 cohorts, 57 smaller ones and 53 of large size were screened (*P* = 0.190). Nonfunctioning pNETs consisted of the major proportion of the cases in which 87.5% and 90.9% were in the grade 1 (*P* = 1.000) and grade 2/3 cohorts (*P* = 0.392), respectively. LNM differed significantly in the grade 2/3 cohort (*P* = 0.003). According to AJCC Staging Classification, there were 53 stage I patients, 116 stage II and 37 stage III in total 206 patients, without stage IV. Of the 206 patients, 68 received pancreaticoduodenectomy, 82 received distal pancreatectomy, 14 received spleen-preserved distal pancreatectomy, 16 received central pancreatectomy, 21 received enucleation, 5 received total pancreatectomy. As for postoperative complications, the main complications were pancreatic leakage, consisting of biochemical leak (n = 24), grade B pancreatic fistula (n = 14) and grade C pancreatic fistula (n = 2). Apart from that, 7 patients experienced delayed gastric emptying, 2 had abdominal abscess and 2 suffered a second operation. No death was found.

**Table 1:** Patient demographics and breakdown based on tumor grade from SHPCI

Characteristic	Patient number (n)	Tumor grade: 1		*P*	Patient number (n)	Tumor grade: 2/3		*P*
		LNM negative (n = 89)	LNM positive (n = 7)			LNM negative (n = 81)	LNM positive (n = 29)	
Age (years)								
< 60	73	67	6	0.871	70	52	18	0.838
≥ 60	23	22	1		40	29	11	
Sex								
Female	45	42	3	1.000	41	30	11	0.932
Male	51	47	4		69	51	18	
Grade								
2	–				98	77	21	0.003
3	–				12	4	8	
Stage								
I	38	38	0	n.a	15	15	0	n.a
II	51	51	0		65	65	0	
III	7	0	7		30	1	29	
IV	0	–	–		0	–	–	
Tumor location								
Head	37	34	3	0.056	50	36	14	0.229
Body/ Tail	57	54	3		57	44	13	
Others	2	1	1		3	1	2	
Size								
1–4 cm	72	68	4	0.497	57	45	12	0.190
> 4 cm	24	21	3		53	36	17	
Functional status								
Nonfunctional	84	78	6	1.000	100	72	28	0.392
Functional	12	11	1		10	9	1	

*n.a.* not available

### Lymph node metastasis was associated with OS

**Fig. 1:**
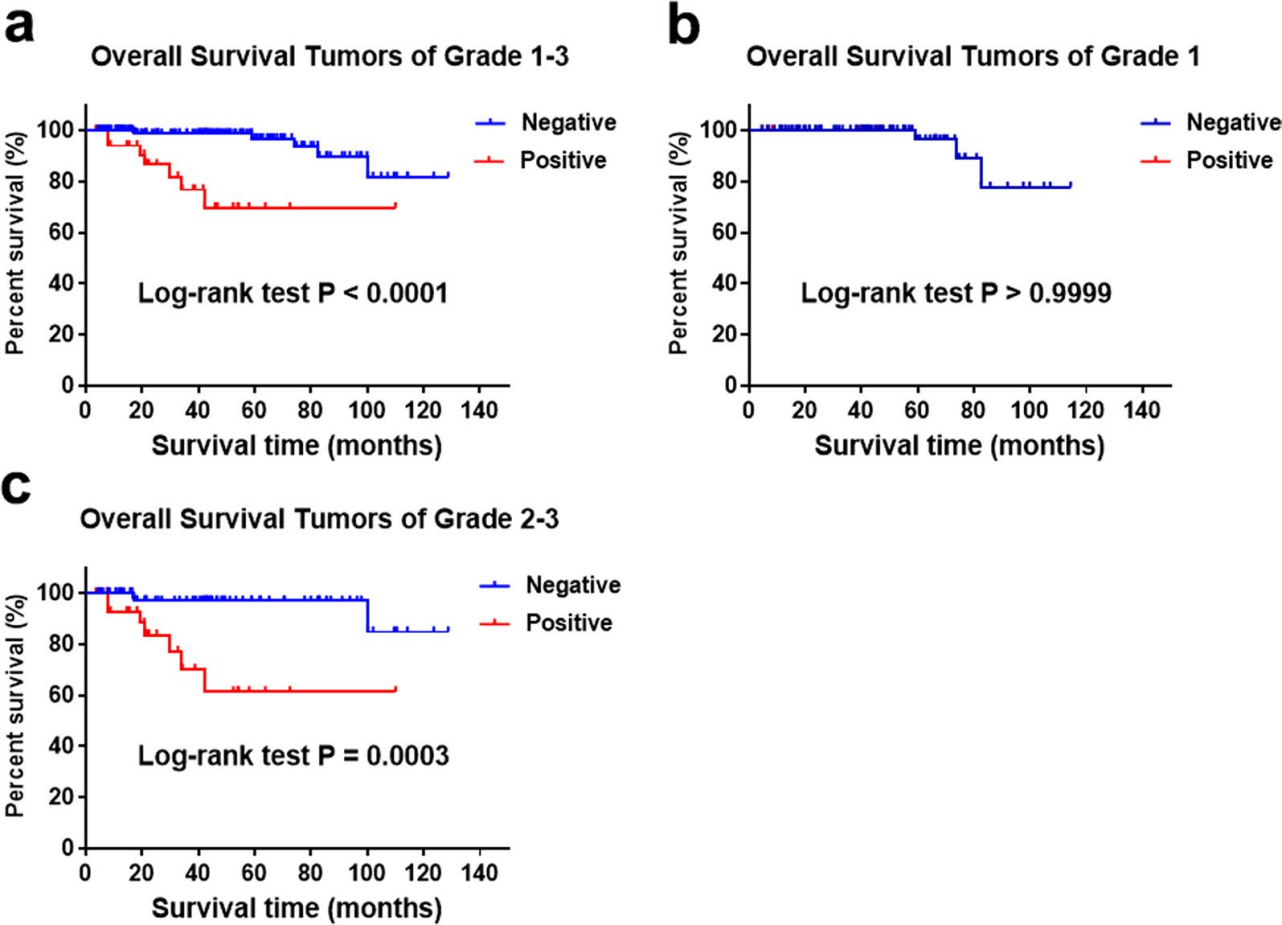
Overall survival for tumors of grade 1–3, grade 1 and grade 2/3 stratified by LNM from SHPCI (**a** log rank < 0.0001; **b** log rank > 0.9999; **c** log rank = 0.0003)

For the 206 patients with pNETs, the LNM was significantly associated with OS (Fig. 1a). In addition, multivariate Cox regression analysis suggested that LNM was an independent prognostic factor for entire cohort (Additional file 1: Table S1). In patients from the single center series with grade 1 tumors, LNM was not substantially associated with OS (Fig. 1b). In patients from the SHPCI series with grade 2/3 tumor, LNM was significantly associated with poorer OS (Fig. 1c).

**Fig. 2:**
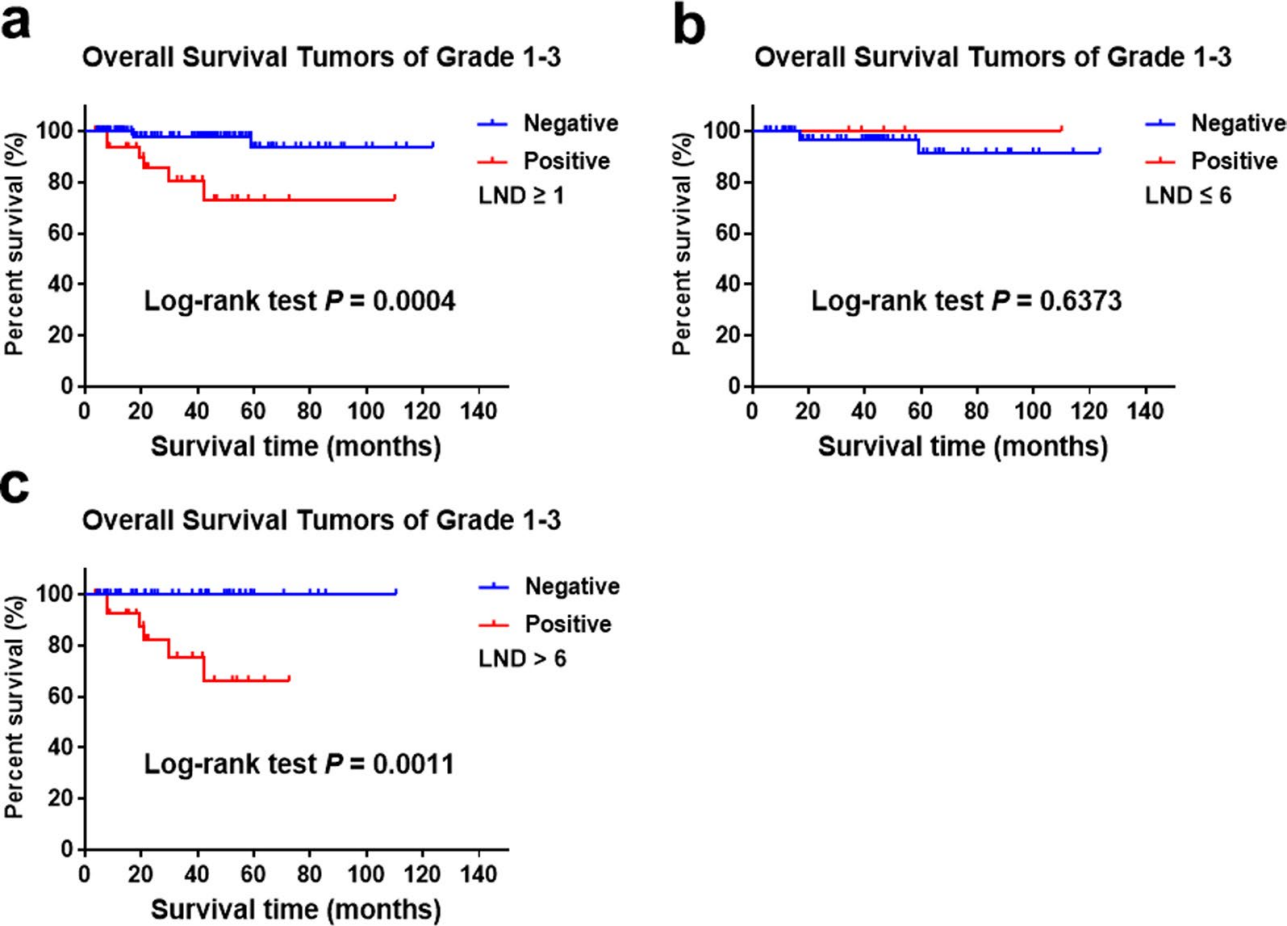
Overall survival for tumors of grade 1–3 stratified by LNM and LND from SHPCI (**a** log rank = 0.0004; **b** log rank = 0.6373; **c** log rank = 0.0011)

**Fig. 3:**
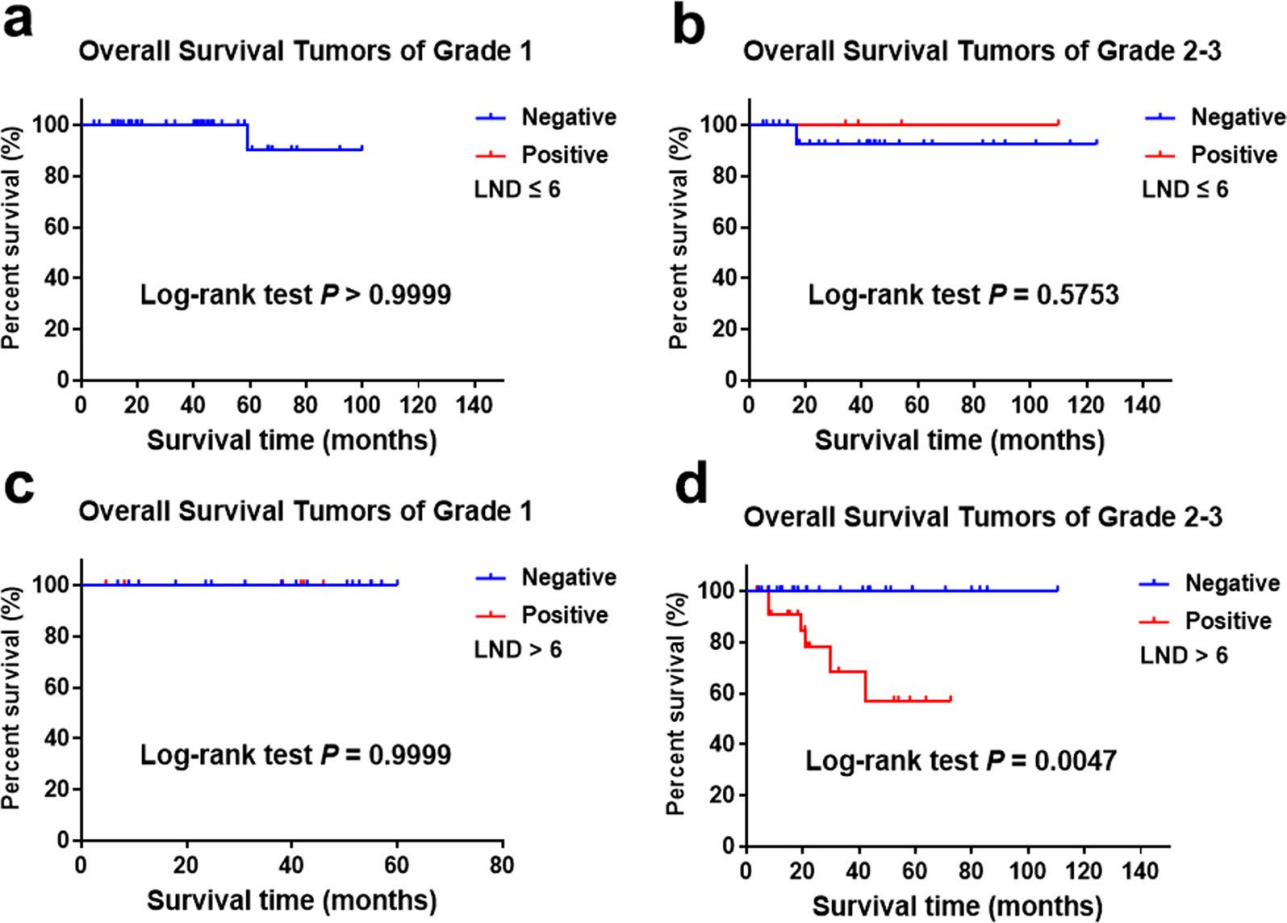
Overall survival for tumors of grade 1 and grade 2/3 stratified by LNM and LND from SHPCI (**a** log rank ≥ 0.9999; **b** log rank = 0.5753; **c** log rank = 0.9999; **d** 0.0047)

Considering the importance of lymph node dissection (LND) for the detection of LNM, we further studied the effect of LNM on prognosis according to the number of LND. There were 155 patients with LND data from the 206 cases and the median LND is 6. The positive rate of LNM was 21.3% in 155 cases in which LNM was significantly correlated with prognosis (Fig. 2a). For patients with LND ≤ 6, the positive rate of LNM was 6.3% in which LNM or not was no longer related to prognosis (Fig. 2b). In patients with LND > 6, the positive rate of LNM was 37.3% in which LNM was significantly correlated with prognosis (Fig. 2c). For patients with LND ≤ 6, LNM was no longer associated with prognosis after stratification according to grade (Fig. 3a and b). However, for grade 1 patients with LND > 6, there was no significant correlation between LNM and OS (Fig. 3c). For grade 2/3 patients with LND > 6, there was a significant correlation between LNM and OS (Fig. 3d).

### Factors predicting OS and LNM

Univariate Cox regression analysis suggested that the LNM was a prognostic factor for OS in the grade 2/3 but not grade 1 cohort (HR, 8.533; 95% CI, 2.165–33.621; *P* = 0.002) (Table 2). Higher grade (*P* < 0.001) and increasing size (*P* = 0.049) were substantially correlated with LNM in patients undergoing nodal harvest (Table 3). When the cohort was further analyzed, for grade 2/3 tumors, higher grade (*P* = 0.002) predicted LNM in the grade 2/3 cohort (Table 3). No factors could predict LNM in grade 1 tumors.

**Table 2:** Cox univariate regression analyses of factors affecting OS by grade from SHPCI

Factor	Tumor Grade: 1		Tumor Grade: 2/3	
	OS		OS	
	HR (95% CI)	*P*	HR (95% CI)	*P*
Age	0.026 (< 0.001–796.339)	0.490	1.396 (0.392–4.971)	0.607
Sex	0.407 (0.036–4.557)	0.466	0.408 (0.115–1.450)	0.166
Tumor location				
Head	1	0.285	1	0.294
Body/tail	0.269 (0.024–2.991)	0.285	0.563 (0.151–2.108)	0.394
Total pancreas	–		3.167 (0.364–27.565)	0.296
Size	6.616 (0.546–80.147)	0.138	0.992 (0.276–3.569)	0.990
Function	0.035 (< 0.001–7644.397)	0.593	0.041 (< 0.001–251.485)	0.472
LNM	–		8.533 (2.165–33.621)	0.002

*OS* overall survival, *HR* hazard ratio, *CI* confidence interval, *LNM* lymph node metastasis

**Table 3:** Binary Regression Analyses of Factor Affecting Lymph Nodal Metastasis Grouped for Grade from SHPCI

Factor	Entire cohort		Tumor grade: 1		Tumor grade: 2/3	
	OR (95% CI)	*P*	OR (95% CI)	*P*	OR (95% CI)	*P*
Age	1.206 (0.502–2.895)	0.676	0.442 (0.040–4.919)	0.507	1.479 (0.545–4.018)	0.442
Sex	1.067 (0.466–2.443)	0.878	1.444 (0.241–8.652)	0.688	1.010 (0.381–2.676)	0.984
Grade						
1	1	< 0.001	–		–	
2	2.799 (1.065–7.352)	0.037	–		–	
3	23.845 (5.420–104.905)	< 0.001	–		8.649 (2.155–34.712)	0.002
Tumor location						
Head	1	0.067	1	0.183	1	0.249
Body/Tail	0.576 (0.251–1.324)	0.194	0.645 (0.115–3.616)	0.618	0.562 (0.211–1.501)	0.250
Total pancreas	5.592 (0.708–44.183)	0.103	16.882 (0.461–618.411)	0.124	3.715 (0.269–51.207)	0.327
Size	2.344 (1.002–5.484)	0.049	2.148 (0.382–12.090)	0.386	2.390 (0.881–6.485)	0.087
Function	0.857 (0.172–4.271)	0.851	1.339 (0.124–14.472)	0.810	0.612 (0.067–5.615)	0.664

*OR* odd ratio, *CI* confidence interval

## Discussion

Recently, systemic treatment of metastatic and advanced pNETs has made progress, although surgical resection remains the only radical therapy and represents the mainstay of treatment for resectable pNET [20–22]. However, pancreatic surgery for pNETs is associated with potential morbidity [23]. Thus, the optimal management for pNET currently remains controversial and the therapeutic strategies range from observation to surgery. The surgery varies from formal resection including pancreaticoduodenectomy or distal pancreatectomy to tumor enucleation with or without lymphadenectomy [23–25]. The NCCN guidelines advocate formal resection with lymphadenectomy in tumors > 2 cm, but there is no firm consensus for smaller tumors. The guidelines suggest radiographic surveillance, formal resection, or enucleation with or without lymphadenectomy in smaller tumors, while lymphadenectomy is recommended for tumors of 1–2 cm in consideration of the risk of LNM [26]. Despite these local surgical procedures historically showing short-term benefits, there is no conclusion on whether these surgical innovations have compromised long-term outcomes because of the indolent nature of these tumors. Given the lack of definitive treatment guidelines, physicians must evaluate multiple factors including LN status when determining the surgical procedure. Several studies have demonstrated that 30–40% of patients with nonfunctional pNET were diagnosed with LNM [27, 28]. Thus, it is important to recognize preoperatively patients at high risk of LNM, who may benefit from lymphadenectomy.

Therefore, we first evaluated whether LNM was correlated with OS. Next, we sought to identify the related factors predicting LNM to guide clinical therapeutic decisions and avoid more aggressive therapies in low-risk patients. We noted that the guidelines may prefer to harvest LND ≥ 12. However, in the real world, surgeons generally remove fewer lymph nodes for neuroendocrine tumors than for pancreatic cancer because of inconsistencies in understanding the importance of lymph node dissection. Therefore, we chose the median number of lymph nodes harvested in our group for grouping in our study. Thus, grouped patients with LNM undergoing harvest LND 1–6 or > 6 lymph nodes.

The results on the prognostic value of LNM in pNET have been conflicting [29, 30]. For example, some researchers have found that patients with LNM have a poor outcome [31, 32], while others have reported that LNM does not decrease survival [33, 34]. Our study indicated that LNM was a predictor of OS in grade 2/3 patients, while there was no such association in grade 1 patients. The data from our centric series confirmed the positive significance of regional lymphadenectomy in grade 2/3 patients, while adequate lymphadenectomy is not recommended for grade 1 patients because LNM shows key prognostic information about survival. Additionally, the univariate analysis suggested that LNM was a prognostic factor for grade 2/3 patients. However, prognostic factors for OS for grade 1 patients did not include LNM. Although LNM or not was no longer related to prognosis for patients with LND ≤ 6, LNM was significantly correlated with a poorer prognosis for patients with LND > 6. Moreover, there was no significant correlation between LNM and OS for grade 1 patients with LND > 6. While there was a significant correlation between LNM and poorer OS for grade 2/3 patients with LND > 6. When LND is sufficient, the conclusion that LNM predicts a poor prognosis for grade 2/3 patients with pNETs is still valid. Currently, the number of harvested lymph nodes for pNETs has not yet been properly addressed. Although, based on pancreatic cancer, it is recommended at least 12 lymph nodes should be removed for pNETs. Most surgeons generally believe that the role of LND is not very important and it is not necessary to expand the dissection to obtain more lymph nodes, so we have some patients with a relatively small number of LND [35]. Therefore, regional lymphadenectomy may not be necessary for grade 1 patients and it is reasonable to make more selective decisions. Patients with more adverse tumor biology may benefit from removing occult nodal diseases.

We found that tumor grade and tumor size were associated with LNM. Given that it is often possible to get these two factors, we mainly focused on the tumor grade and size, which can be determined before surgery. Aguiar et al. found an increased prevalence of LNM in nonfunctional pNETs > 2 cm. However, they reported that 9% of patients with tumor size < 2 cm had LNM [36, 37]. In contrast, Parekh et al. found that tumor size could not significantly predict LNM, although 31% of patients with LNM had tumors < 3 cm [29]. Haynes et al. reported that factors positively associated with progression or metastasis of the disease also included tumor size (> 2 cm). However, among patients with tumor size < 2 cm, 8% of patients had metastasis [38]. Our data indicated that tumors > 4 cm were almost twofold as likely to have LNM compared with tumors < 4 cm. However, 12.4% of patients with 1–4 cm tumors had LNM.

Grade 1 was correlated with a significantly low risk of LNM. Additionally, we found that LNM reliably predicted OS based on grade. Thus, clinical decisions may benefit from the classification of tumor grade, which usually depends on accurate pathological examination. Consequently, preoperative pathological evaluation can be performed using EUS combined with FNA. Piani et al. reported that Ki-67 expression on histological sections had good agreement with Ki-67 expression measured in cytological samples after EUS, in which the Ki-67 value was consistent in 89% and 78% of patients for Ki-67 values of 2% and of 2%–10%, respectively [39]. Hasegawa et al. reported a 90% concordance rate for surgical histopathology with EUS–FNA-evaluated tumor grade using > 2000 cells (74% of patients) [40]. The high concordance and reproducibility of EUS–FNA-determining Ki-67 values were further demonstrated by Weynand and colleagues [41]. EUS–FNA is usually performed only at highly experienced centers. Preoperative examination of tumor grade, combined with tumor size may guide surgeons to choose the best surgical procedure and whether regional lymphadenectomy should be performed.

Additionally, regional lymphadenectomy may lead to the inclusion of splenectomy, increased blood loss, longer operating time and hospital stay, and increased lymphocele development. Thus, the benefits and risks of lymphadenectomy should be evaluated carefully. Our study demonstrated that there was no difference in OS between grade 1 patients with LNM and those without LNM. The benefits of lymphadenectomy in patients with grade 1 tumors remain unclear and more clinical trials and high-quality clinical data are needed to deal with the problem. Moreover, it is not clear if lymphadenectomy should be omitted for small nonfunctional pNETs because of low rates of LNM, and better prognosis compared with larger tumors. Gratian et al. reported that whether lymphadenectomy was performed did not significantly affect the 5-year OS in 1854 operated patients with nonfunctional pNETs ≤ 2 cm [33]. Rui Mao et al. reported that lymphadenectomy did not show any survival benefit in patients undergoing resection for pNETs [27]. Based on the current research on LND, many surgeons routinely carry out functional sparing surgery, such as spleen preservation, enucleation, middle pancreatectomy and so on [27, 32]. Such surgical decision-making mainly depends on the size of the tumor in which functional sparing surgery is generally chosen for smaller tumors. However, there is still a certain risk in oncology. The present study revealing that the LNM was not associated with the prognosis of patients with grade 1 pNETs may provide some theoretical basis. Lymphadenectomy may not be performed routinely in patients with grade 1 and/or small tumors.

There were several limitations to the present study that should be considered in the interpretation of the data. First, the data collected from the SHPCI series were retrospective, thus well-designed clinical trials need to be performed to verify the results. Second, the database is from a single center and the sample size is not very large, therefore, the subgroup analysis was not sufficient. In addition, the low amount of LNM seen in grade 1 (which is expected with lower-grade tumors) may not provide enough power for survival. Finally, some patients from the SHPCI series lacked detailed follow-up data including postoperative imaging or care.

## Conclusions

Based on our own retrospective data obtained from a single center series, LNM seems to be associated with poorer outcome for patients with grade 2/3 and/or grade 1 > 4 cm tumors. On the other way, LNM was seems to be not associated with prognosis in patients with grade 1 tumors less than 4 cm. Moreover, tumor grade and tumor size seem to act as independent predictors of LNM. Thus, regional lymphadenectomy should be performed in grade 2/3 patients but was not mandatory in grade 1 tumors < 4 cm. It is reasonable to perform functional sparing surgery for grade 1 patients or propose a clinical-radiological monitoring.

## Supplementary Information

**Additional file 1.** Cox Multivariate Regression Analyses of Factors Affecting OS from SHPCI.

## Data Availability

All data generated or analysed during this study are included in this published article.

## Abbreviations

pNET: Pancreatic neuroendocrine tumor
LNM: Lymph node metastasis
SHPCI: Shanghai pancreatic cancer institute
OS: Overall survival
AJCC: American Joint Committee on Cancer
ENETS: European Neuroendocrine Tumor Society
NCCN: National Comprehensive Cancer Network
DFS: Disease-free survival
HRs: Hazard ratios
CIs: Confidence intervals
LND: Lymph node dissection
