# Supplementary material for: Value of lymphadenectomy in patients with surgically resected pancreatic neuroendocrine tumors
Source: BMC Surg. 2022 May 10;22:160. doi: 10.1186/s12893-022-01595-y (PMC9088076; doi:10.1186/s12893-022-01595-y)
Supplement: Supplementary file 1 — Additional file 1. Cox Multivariate Regression Analyses of Factors Affecting OS from SHPCI. [file 12893_2022_1595_MOESM1_ESM.docx]

| **Table S1 Cox Multivariate Regression Analyses of Factors Affecting OS from SHPCI** | | |
| --- | --- | --- |
| **Factor** | **Entire cohort** | |
|  | **OS** | |
|  | **HR (95% CI)** | ***P*** |
| **Age** | 0.911 (0.266-3.116) | 0.881 |
| **Sex** | 0.514 (0.160-1.646) | 0.262 |
| **Tumor**  **location** |  |  |
| **Head** | 1 | 0.407 |
| **Body/tail** | 0.449 (0.132-1.532) | 0.201 |
| **Total pancreas** | 1.188 (0.128-11.068) | 0.880 |
| **Size** | 0.624 (0.158-2.460) | 0.500 |
| **Function** | **-** | 0.984 |
| **Grade** | 2.052 (0.466-9.043) | 0.342 |
| **LNM** | 7.055 (1.912-26.031) | 0.003 |

OS: Overall survival; HR: hazard ratio; CI: Confidence interval; LNM: lymph node metastasis
